# Idiopathic superior mesenteric vein thrombosis leading to gangrenous ileum: A case report

**DOI:** 10.1016/j.ijscr.2025.111561

**Published:** 2025-06-24

**Authors:** Samrat Shrestha, Kiran Bishwakarma, Bijay Raj Bhatta, Mecklina Shrestha, Rahul Jha

**Affiliations:** a National Academy of Medical Sciences, NAMS, Bir Hospital, Department of General Surgery, Kathmandu, Nepal; b College of Medical Sciences(CoMS), Department of Pathology, Bharatpur, Nepal

**Keywords:** Superior mesenteric vein, Thrombosis, Gangrenous ileum, Emergency laparotomy, Anticoagulation, Case report

## Abstract

**Introduction and importance:**

Idiopathic superior mesenteric vein thrombosis (SMVT) is an uncommon but serious cause of mesenteric ischemia, accounting for 6–9 % of cases. Although SMVT is typically linked to hypercoagulable states, malignancy, or intra-abdominal inflammation, an idiopathic etiology persists in up to 25 % of cases even after exhaustive evaluation, underscoring diagnostic challenges and the need for vigilant workup.

**Case presentation:**

A 46-year-old male presented with progressive abdominal pain, distension, and bilious vomiting. CECT identified SMVT with non-enhancing ileal loops, confirming gangrenous ileum. Emergency laparotomy revealed 100 cm of necrotic ileum, which was resected with ileostomy creation. Postoperative anticoagulation was initiated, and thrombophilia screening ruled out hypercoagulable disorders, classifying the SMVT as idiopathic.

**Clinical discussion:**

SMVT presents insidiously with abdominal pain, vomiting, and distension; late features include peritonitis and hemodynamic instability. CECT remains the diagnostic gold standard with >90 % accuracy. Management algorithms stratify patients by stability and ischemia severity: stable patients may respond to anticoagulation alone, while those with infarction require prompt surgical resection and possible stoma formation. Lifelong or extended anticoagulation is advocated in idiopathic cases to prevent recurrence.

**Conclusion:**

This case underscores that idiopathic SMVT, although rare and often diagnostically challenging, can rapidly progress to life-threatening bowel gangrene even in the absence of known risk factors. Timely diagnosis using contrast-enhanced CT and a multidisciplinary approach—including prompt surgical intervention and postoperative anticoagulation—are critical to improving survival and reducing long-term morbidity. This case reinforces the importance of maintaining a high index of suspicion and implementing early, coordinated management in suspected mesenteric ischemia.

## Highlights

• Idiopathic SMVT is a rare, life-threatening condition causing bowel ischemia, demanding high clinical suspicion for diagnosis.
• CECT is the gold standard for diagnosing SMVT, revealing thrombus, bowel wall thickening, and signs of transmural infarction.
• Conservative management with anticoagulation is effective in stable cases, while surgical resection is vital in infarction cases.
• Prompt surgical intervention, including bowel resection and ileostomy, prevents complications from progressing bowel necrosis.
• Lifelong anticoagulation may be necessary in idiopathic SMVT to reduce recurrence risk and avoid long-term thrombotic events.

## 1 Introduction

Superior mesenteric vein thrombosis (SMVT) is a rare yet potentially life-threatening condition that leads to mesenteric ischemia and bowel infarction if not diagnosed and treated promptly. It accounts for approximately 6–9 % of cases of mesenteric ischemia, with arterial thrombosis being more common [1]. The condition is often associated with underlying disorders that disrupt Virchow's triad: hypercoagulability, stasis, and endothelial injury [2]. Idiopathic SMVT is an even rarer condition, diagnosed after a meticulous search for the thrombosis-causing pathology, which is responsible for 25 % of the cases [1]. In idiopathic SMVT, proposed pathophysiological mechanisms include transient prothrombotic states, occult endothelial injury, and localized venous stasis, yet many cases remain unexplained despite extensive workup.[3] Differential diagnoses include acute arterial mesenteric ischemia, small bowel obstruction, and intra-abdominal inflammatory conditions such as pancreatitis, which can present with overlapping clinical features, necessitating careful evaluation.[4] Imaging studies, particularly contrast-enhanced computed tomography (CECT), play a pivotal role in diagnosis. CECT findings suggestive of SMVT include circumferential bowel wall thickening, vascular changes, and nonmural/nonvascular signs [5]. Management strategies for SMVT vary based on the severity of the condition and the patient's overall health. Conservative treatment with anticoagulation therapy is often effective in stable patients [6]. However, in cases of bowel infarction or peritonitis, surgical intervention is necessary, which may involve bowel resection and the creation of an ileostomy to manage intestinal continuity [7]. Given the rarity and potential severity of SMVT, a high index of suspicion is essential for early diagnosis and effective management. We present a case of idiopathic SMVT to highlight diagnostic intricacies and the role of timely intervention. This case has been reported in line with the revised SCARE guidelines, 2025 [8].

## 2 Case presentation

**Fig. 1:**
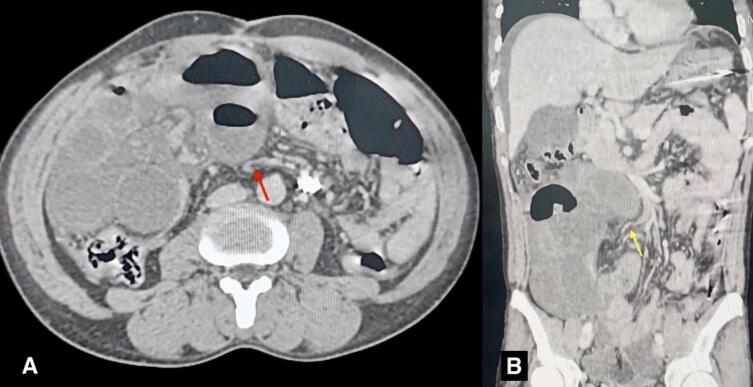
CECT scans axial (A) and coronal (B) sections showing a filling defect in the branch of the superior mesenteric vein (SMV) indicated by a red arrow in the axial section and a yellow arrow in the coronal section. CECT: Contrast-enhanced computed tomography. (For interpretation of the references to color in this figure legend, the reader is referred to the web version of this article.)

A 46-year-old male presented to the emergency department of our hospital with a 4-day history of generalized abdominal pain and distension, accompanied by multiple episodes of bilious vomiting. The symptoms had progressively worsened, prompting him to seek medical attention. The patient had no significant past medical or surgical history. There was no family history of any thrombotic events. The patient was a nonsmoker. On arrival, the patient was tachycardic, with a pulse rate of 110 beats per minute and blood pressure of 100/70 mm Hg, temperature was 37.8 °C, respiratory rate of 22 breaths per minute, and oxygen saturation 95 % on room air. The patient was alert and oriented. Physical examination revealed dehydration, abdominal distension, and generalized tenderness on abdominal palpation. Laboratory parameters were hemoglobin: 12.7 g/dl (normal: 14–18 g/dl); total leukocyte count: 12,820/μL (normal level: 4000–11,000/μL) with neutrophils of 91 % and lymphocytes of 2 %; random blood sugar: 9.48 mmol/L (normal level: 4.4–7.8 mmol/L); prothrombin time (PT): 22.1 s (normal level: 11–13.5 s); and INR (International Normalized Ratio): 1.54 (normal level: 0.8–1.2). Liver function tests (ALT/AST) were within normal limits, excluding hepatic dysfunction. The rest of the laboratory parameters were within normal limits. Ultrasonography (USG) of the abdomen showed dilated bowel loops and minimal pelvic fluid collection. A CECT scan showed a filling defect in a branch of the superior mesenteric vein (ileal vein), consistent with SMVT, leading to veno-occlusive mesenteric ischemia with the absent enhancement of ileal loops, indicating ischemic changes of the ileum (Fig. 1). Initial management included fluid resuscitation for hemodynamic stabilization and nasogastric decompression. Broad-spectrum antibiotics were initiated to manage the risk of sepsis. Due to generalized peritonitis and bowel ischemia, anticoagulation therapy was withheld, and an emergency exploratory laparotomy was performed. Thrombectomy was not attempted due to diffuse thrombosis and anticipated irreversible gangrene.

**Fig. 2:**
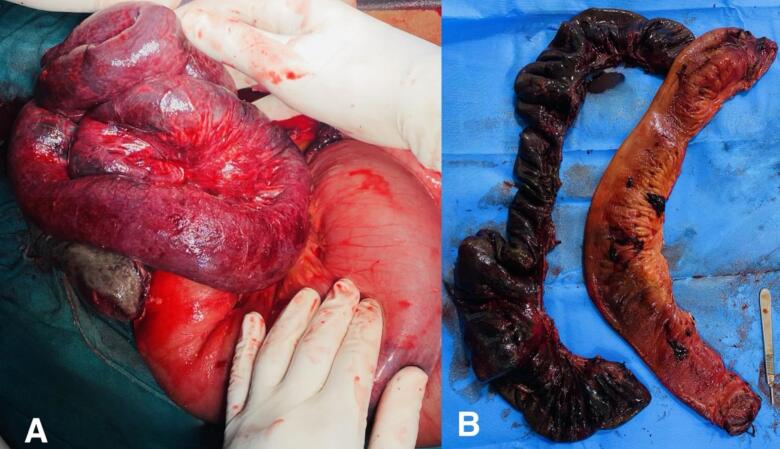
Intraoperative picture showing A. Grossly dilated small and large bowel loops with gangrenous ileal loops. B. Resected specimen of gangrenous ileum.

Intraoperatively, approximately 500 ml of hemorrhagic collection was present in the peritoneal cavity. 100 cm of the gangrenous ileal segment was identified, located 30 cm proximal to the ileocecal junction (Fig. 2). The gangrenous segment was resected, a double-barrel ileostomy was created, and an abdominal drain was kept. Postoperatively, the patient was shifted to the intensive care unit. The patient was started on empirical broad-spectrum intravenous antibiotics upon admission, including piperacillin-tazobactam 4.5 g IV every 8 h and metronidazole 500 mg IV every 8 h, targeting gram-negative and anaerobic organisms. Postoperatively, antibiotics were continued for 5 days based on intraoperative findings and clinical response. Anticoagulant therapy with intravenous heparin was initiated on the 2nd postoperative day (POD). At the 2nd POD, the ileostomy was functional and oral feeding was started. The abdominal drain was removed on the 7th POD. We investigated the cause of the thrombosis during the hospital stay of the patient. We consulted with the hematology department, and a thrombophilia screen for antithrombin III, anti-cardiolipin, anti-B2 glycoprotein, protein C and protein S deficiencies, factor V Leiden mutation, and paroxysmal nocturnal hemoglobinuria was done; all results were negative. A colonoscopy ruled out inflammatory bowel disease (IBD). Given the negative work-up, a diagnosis of idiopathic SMVT was established.

The patient was discharged on the 14th POD with an oral factor Xa inhibitor, rivaroxaban, for 6 months. Here direct oral anticoagulant was chosen over vitamin K antagonists because of its better patient compliance with easy dosing and avoidance of frequent coagulation profiling. He remained asymptomatic on 2-week, 1-month, and 2-month follow-ups, with no recurrence of thrombosis. Stoma reversal was done after 2 months. Subsequent follow-up for 6 months showed no signs of recurrence. This case highlights the critical importance of early identification and surgical intervention in managing superior mesenteric vein thrombosis to prevent fatal outcomes associated with bowel ischemia.

This case presented multiple challenges, beginning with a vague and nonspecific clinical picture that led to a delay in diagnosis. The absence of common risk factors for SMV thrombosis complicated the diagnostic process, and limited access to advanced imaging in our setting initially hindered prompt evaluation. Intraoperatively, the extent of bowel ischemia was greater than anticipated, necessitating careful judgment in determining the resection margin. Additionally, managing postoperative anticoagulation in the absence of an identifiable etiology posed further difficulty. The patient described the event as profoundly life-altering but expressed gratitude for timely intervention. Post-recovery, he emphasized ‘learning to recognize early warning signs’ and committed to lifelong vigilance despite anticoagulation cessation, stating ‘this experience made me prioritize health awareness’.

## 3 Discussion

Mesenteric vein thrombosis is a rare but potentially fatal disease process, characterized by the formation of a clot within the superior or inferior mesenteric vein or its branches. Acute SMVT accounts for 6–9 % of all cases of acute mesenteric ischemia [1]. The common causes of SMVT include hypercoagulable states (Antiphospholipid antibodies, Deficiencies of Antithrombin III, protein C or S, pregnancy, OCPs), malignancy, recent surgery, inflammatory conditions (pancreatitis, inflammatory bowel disease), portal hypertension, etc. Suppurative pylephlebitis is another rare cause of SMVT, mostly following acute appendicitis or its sequelae, acute diverticulitis, and intra-abdominal abscess [2]. Idiopathic SMVT is termed when all the known causes of SMVT have been searched and ruled out. While >25 % of cases of SMVT may initially appear idiopathic, with thorough examinations and investigations, many of them will have an identifiable cause [1]. Common identifiable causes after exhaustive investigations include occult malignancies, myeloproliferative disorders, autoimmune diseases, inflammatory bowel diseases, immune thrombocytopenic purpura, portal hypertension, and liver cirrhosis [1,9].

Longitudinal studies reveal that 25–40 % of initially idiopathic SMVT cases are later reclassified after extended surveillance. The most frequently uncovered etiologies include: Occult malignancies (particularly pancreatic/colorectal adenocarcinomas and lymphomas), missed due to sub radiologic tumor burden at initial presentation [10]. In myeloproliferative neoplasms (e.g., JAK2 V617F+ essential thrombocythemia), thrombotic events often precede hematologic abnormalities by years [11]. And in seronegative autoimmune disorders (e.g., antiphospholipid syndrome), the antibody titers may fluctuate below diagnostic thresholds [12].

SMVT can be divided into acute, subacute, or chronic types based on the presentation. In acute SMVT, there will be a colicky type of severe mid-abdominal pain lasting for a few hours, with a risk of bowel infarction and peritonitis. Our case is of acute type. In subacute cases, bowel infarction is less likely though there is associated severe abdominal pain. Whereas in chronic SMVT, because of extensive collateral circulation, there will rarely be any pain [13]. Apart from the specific presenting features of the underlying etiology, the common clinical features of SMVT include diffuse abdominal pain, nausea or vomiting, anorexia, and abdominal distention. Because of its nonspecific clinical signs and symptoms, its early diagnosis and treatment seem difficult, leading to high morbidity and mortality. Features of generalized peritonitis, hemodynamic instability, and fever are the late findings in SMVT [4]. Bowel ischemia in mesenteric vein thrombosis is because of blockage of venous blood flow resulting in increased capillary hydrostatic pressure, which then results in a series of changes that include bowel wall edema, arterial inflow obstruction, and ultimately bowel wall infarction [1].

Plain abdominal radiographs may reveal dilated bowel loops with an air-fluid level, thumbprint sign, and free air in case of bowel perforation. The next best approach for non-specific abdominal pain with suspicion of SMVT would be CECT of the abdomen and pelvis, with a diagnostic accuracy of >90 % [5]. Features on CECT scans include a filling defect within the superior mesenteric vein or its branches, mesenteric congestion, and stranding, bowel wall thickening, and in some cases, ascites. Bowel wall thickening (often >3 mm) appears as hypo-attenuating due to edema, and in some cases, there may be air in the bowel wall (pneumatosis intestinalis) because of transmural infarction [14]. Other imaging modalities, such as Magnetic Resonance Imaging (MRI), angiography, and abdominal Doppler ultrasonography, are less commonly preferred tools and can be considered as second-line modalities in cases of diagnostic dilemma [5,14]. Another diagnostic tool in case of uncertainty of the underlying disease process is diagnostic laparoscopy, which then be continued as a therapeutic procedure or converted to open laparotomy based on patient factors and surgeon preference [15]. In this case, the patient presented with signs of intestinal obstruction—diffuses abdominal pain, bilious vomiting, and absence of flatus or bowel movements—along with clinical features of peritonitis, such as guarding and absent bowel sounds. These findings, combined with leukocytosis and elevated inflammatory markers, raised a strong suspicion of bowel ischemia. The absence of prior comorbidities or precipitating factors initially obscured the diagnosis, but imaging confirmed SMVT with gangrenous changes. In our case, early CECT was pivotal in identifying the thrombus and guiding surgical decision-making.

After the diagnosis of SMVT has been made, treatment planning should be done based on the timing and severity of the presentation, side by side, looking for the etiology. For that, recommended laboratory studies include a complete blood count, coagulation tests, a thrombophilia screen, and a comprehensive metabolic panel [3]. The multidisciplinary management of superior mesenteric vein thrombosis (SMVT) hinges on early diagnosis (contrast-enhanced CT), immediate anticoagulation for stable patients, and urgent surgery if bowel necrosis or peritonitis is suspected. A collaborative approach involving emergency surgeons, gastroenterologists, interventional radiologists, and hematologists is crucial—ensuring proper anticoagulation, addressing underlying causes (e.g., thrombophilia or cirrhosis), and considering endovascular or surgical interventions when needed. Long-term anticoagulation is often required to prevent recurrence, guided by hematological assessment. The key is balancing medical therapy with timely surgical intervention to optimize outcomes [16].

For a stable patient with early presentation, treatment options include resuscitation, bowel rest, and anticoagulation or thrombolytic therapy. Anticoagulation therapy using either intravenous unfractionated heparin (UFH) or subcutaneous low-molecular-weight heparin (LMWH) is the treatment modality of choice in patients with milder symptoms [6]. After resolution of the symptoms, anticoagulation therapy is switched to the oral form (factor Xa inhibitor or vitamin K antagonist), which is continued on a long-term basis (usually for 3–6 months; sometimes, lifelong) according to the European Society for Vascular Surgery (ESVS) guidelines, 2017 [17]. More severe presentation or failure of anticoagulation therapy warrants the need for endovascular thrombolytic therapy by pharmacological means or balloon angioplasty [18]. Complications following thrombolytic therapy, failure of thrombolysis, or the presence of signs of bowel infarction or perforation at the time of presentation are indications of exploratory laparotomy. After a midline laparotomy, an assessment of the bowel viability should be done, which can be aided using intraoperative Doppler ultrasound or by indocyanine green fluorescence test. Open thrombectomy of the affected vein, followed by repair, helps in the revascularization of the affected bowels [19]. In case of gangrenous bowel loops or perforation, resection of the necrotic tissues should be done, followed by restoration of the bowel continuity by anastomosis. Sometimes a diversion stoma is created in case of gross contamination or hugely dilated, edematous bowel walls. In a few cases, a re-look laparoscopy or laparotomy is needed to reassess for additional ischemic bowel segments [7]. Another effective way of managing SMVT is by hybrid techniques, using a combination of endovascular and open surgical approaches [20]. In our case, thrombolysis was not attempted due to generalized peritonitis and anticipated bowel ischemia; hybrid approaches were unavailable emergently.

Long-term surveillance is crucial for detecting complications and recurrence. Patients with extensive resections (>50–100 cm) are at risk for short bowel syndrome (SBS), presenting with malnutrition, electrolyte imbalances, and dependency on parenteral nutrition [21]. Regular monitoring includes nutritional assessments (albumin, micronutrients), abdominal imaging, and thrombophilia reevaluation [3,7]. In this case, 100 cm ileal resection did not cause SBS due to preserved jejunum and ileocecal valve but required biannual B12/folate checks. Lifelong clinical vigilance remains essential as recurrence can occur years after anticoagulation cessation [17,21].

In idiopathic SMVT, the risk of recurrence is substantial, estimated at 20–30 % without extended anticoagulation [3]. This underscores the need for tailored anticoagulation strategies. While guidelines recommend lifelong anticoagulation for idiopathic cases, we opted for a 6-month course of rivaroxaban in this patient [17]. This decision balanced recurrence risk against bleeding concerns, considering his young age, absence of thrombophilia, surgical resection of the infarcted segment (eliminating the nidus for thrombosis), and potential transient prothrombotic triggers. Close long-term surveillance was prioritized to detect recurrence early, acknowledging that therapy duration remains individualized [17].

Postoperative care intensity depends on the etiology of SMVT and the severity of the patient at the time of presentation. During the initial post-operative days, care should be given to early identification of redevelopment of thrombosis, progression of bowel ischemia, and anastomosis/ostomy-related complications. One of the important surgery-related complications is the development of short bowel syndrome (SBS) [21]. Anticoagulation therapy should be continued postoperatively along with appropriate coagulation panel screening. Lifelong anticoagulation may be needed in case of idiopathic etiology [17].

## 4 Conclusion

SMVT is a rare and potentially life-threatening cause of mesenteric ischemia that demands prompt diagnosis and intervention to prevent severe complications such as bowel infarction and peritonitis. This case emphasizes the importance of early recognition through clinical evaluation and advanced imaging, such as CECT, in diagnosing SMVT. The patient's rapid progression to generalized peritonitis highlighted the need for urgent surgical intervention, including resection of the gangrenous ileal segment and the creation of a double-barrel ileostomy. These timely interventions, coupled with post-operative management, including anticoagulation therapy, are essential to minimize the risk of recurrence and to optimize long-term outcomes. This case underscores the critical role of a multidisciplinary approach, integrating surgical, medical, and radiological expertise, in the effective management of SMVT and highlights the need for early intervention to prevent the high mortality associated with delayed treatment.

## CRediT authorship contribution statement

1. Constructing a hypothesis for the manuscript- Samrat Shrestha, Kiran Bishwakarma, Bijay Raj Bhatta.
2. Planning methodology to reach the conclusion: Samrat Shrestha, Kiran Bishwakarma, Mecklina Shrestha.
3. Organizing and supervising the course of the article and taking responsibility: Samrat Shrestha.
4. Patient follow-up and reporting – Mecklina Shrestha, Bijay Raj Bhatta, Rahul Jha.
5. Logical interpretation and presentation of the results- Samrat Shrestha, Kiran Bishwakarma, Bijay Raj Bhatta, Mecklina Shrestha, Rahul Jha.
6. Construction of the whole or body of the manuscript- Samrat Shrestha, Bijay Raj Bhatta, Kiran Bishwakarma, Mecklina Shrestha, Rahul Jha.
7. Reviewing the article before submission not only for spelling and grammar but also for its intellectual content-Samrat Shrestha, Bijay Raj Bhatta, Mecklina Shrestha, Kiran Bishwakarma, Rahul Jha.

## Patient consent

Written informed consent for publication of their clinical details and images was obtained from the patient. A copy of the consent is available for review by the Editor-in-Chief of this journal.

## Ethical approval

Ethical approval is not required for case reports in our institution. Patient consent is the primary gateway for the publication of case reports. A written informed consent from the patient has been attached for the publication of data and images.

## Guarantor

The guarantor is Samrat Shrestha.

## Funding statement

The authors declared that no grants were involved in supporting this work.

## Declaration of competing interest

The authors have no conflict of interest to declare.

## Figures and Tables

**Table:** 

Timepoint	Event	Details
Day –4	Symptom onset	Generalized abdominal pain and distension; multiple episodes of bilious vomiting
Day 0 (presentation)	Emergency department presentation	Tachycardia (110 bpm), BP 100/70 mm Hg, dehydration, distension, generalized tenderness
Day 0	Initial imaging	USG: dilated bowel loops, minimal pelvic fluid. CECT: filling defect in ileal branch of SMV; non-enhancement of ileal loops
Day 0	Operative management	Emergency exploratory laparotomy; resection of 100 cm gangrenous ileum; double-barrel ileostomy; drain placement
POD 2	Anticoagulation & oral feeding	Started IV heparin; ileostomy functional; began oral feeding
POD 7	Drain removal	Abdominal drain removed
POD 14	Discharge	Discharged on oral rivaroxaban for 6 months
2-Week follow-up	Outpatient review	Asymptomatic, no recurrence of symptoms
1-Month follow-up	Outpatient review	Asymptomatic, stoma functioning well
2-Month follow-up	Stoma reversal	Reversal of ileostomy
6-Month follow-up	Long-term outcome	No signs of recurrent thrombosis or complications

## Data Availability

All figures and tables included in this case report are original and were created by the authors from their direct source data. No external references or secondary sources were used in the preparation of these figures and tables. The data presented are based solely on the authors' observations and analysis.
